# Evaluation of eco-environmental quality for the coal-mining region using multi-source data

**DOI:** 10.1038/s41598-022-09795-5

**Published:** 2022-04-22

**Authors:** Huan Jiang, Gangwei Fan, Dongsheng Zhang, Shizhong Zhang, Yibo Fan

**Affiliations:** School of Mines, China University of Mining & Technology, No.1 University Road, Xuzhou, 221116 Jiangsu China

**Keywords:** Environmental impact, Sustainability

## Abstract

The contradiction between the exploitation of coal resources and the protection of the ecological environment in western China is becoming increasingly prominent. Reasonable ecological environment evaluation is the premise for alleviating this contradiction. First, this paper evaluates the eco-environment of Ibei coalfield by combining the genetic projection pursuit model and geographic information system (GIS) and using remote sensing image data and other statistical data of this area. The powerful spatial analysis function of GIS and the advantages of the genetic projection pursuit model in weight calculation have been fully used to improve the reliability of the evaluation results. Furthermore, spatial autocorrelation is used to analyze the spatial characteristics of ecological environment quality in the mining area and plan the specific governance scope. The geographic detector is used to determine the driving factors of the eco-environment of the mining area. The results show that Ibei Coalfield presents a spatially heterogeneous eco-environment pattern. The high-intensity mining area (previously mined area of Ili No.4 Coal Mine) has the worst ecological environment quality, followed by the coal reserve area of Ili No.4 Coal Mine and the planned survey area of Ili No.5 Coal Mine. The eco-environment quality (EEQ) of the study area is affected by both human and natural factors. Mining intensity and surface subsidence are the main human factors affecting the ecological environment in the study area. The main natural factors affecting the ecological environment in the study area are annual average precipitation, elevation, annual average evaporation, NDVI and land use type. Meanwhile, the interaction effect of any two indicators is greater than that of a single indicator. It is also indicated that the eco-environment of the mining area is nonlinearly correlated to impact indicators. The spatial autocorrelation analysis shows three areas that should be treated strategically that are the management area, close attention area and protective area. Corresponding management measures are put forward for different regions. This paper can provide scientific references for mining area eco-environmental protection, which is significant for the sustainability of coal mine projects.

## Introduction

Human activities are closely tied to ecology and the environment. Understanding and assessing the eco-environment level is not only an important research focus in the field of energy and environment, but also critically needed by the sustainable economy and civilisation^1^. Coal is a key component of China's energy structure, accounting for 60% of the total annual energy consumption^2^. It has been identified that long-term and high intensive underground mining can lead to a series of problems such as surface subsidence, water level drawdown and vegetation deterioration, further affecting localized ecology and environment^3,4^. Reasonable evaluation of eco-environment in mining area is helpful to analyze the sustainable development of mining area and put forward corresponding countermeasures for eco-environment management in mining area^5^.

The evaluation of the eco-environment is based on the development characteristics of the ecosystem, mainly based on ecological carrying capacity model^6^, landscape pattern model^7^ and remote sensing ecological index model (RSEI)^8^, to build different eco-environmental assessment systems to evaluate specific regions. Ecological carrying capacity refers to the support and capacity of regional resources and environment to social economy and human activities, the most representative methods are energy value analysis, ecological footprint method and life cycle assessment (LCA). Although the energy value analysis method can convert the indexes of different types and units of measurement into the same standard energy value for analysis, it is difficult to put forward the threshold value of the evaluation system^9^. The ecological footprint method is simple and feasible in the evaluation of the ecological environment, but it could not fully consider the impact of human technological progress and social development^10^. LCA can be used to evaluate the capacity of the ecosystem coordinated development. However, it is difficult to confirm the dividing standard of the starting and ending points of each stage of the system life cycle^11^. Landscape pattern models can reflect the instrumental value of the ecosystem services through different landscape types, but the selection of landscape pattern indicators is hard to reflect the spatial heterogeneity and complexity of landscape pattern evaluation^12^. In recent years, the development of remote sensing technology provides a new model for ecological environment assessment. Remote sensing (RS) technology has the characteristics of simple information acquisition and large coverage. Xu et al.^13^ proposed a remote sensing ecological index (RSEI) based on RS data and integrated multiple ecological factors. RSEI uses four environmental elements (greenness, moisture, dryness, heat) to reflect the ecological environment of the region, and the results obtained are objective and stable. However, the indicators selected by RSEI are inadequate and the assessment results do not fully reflect the ecological conditions of the region.

When choose eco-environmental assessment indicators in mining areas, the most commonly used indicators include normalized difference vegetation index (NDVI)^14^, vegetation coverage (VFC)^15^ and soil adjusted vegetation index (SAVI)^16^. However, the supervision and evaluation of a single indicator can only explain the characteristics of the eco-environment in one aspect, which cannot truly and comprehensively reflect the EEQ of the whole mining area. Wang et al.^17^ evaluated the eco-environment of the Gongyi mining area by combining natural factors, climate factors and mineral types. It's based on the factors of the geo-ecological environment and social development, Saedpanah and Amanollahi^18^ used the analytic hierarchy process (AHP) to construct the eco-environment evaluation system of the Qhorveh mining area. However, these comprehensive evaluation methods also have some shortcomings, such as not considering the mining type, mining intensity of and the significant negative impact of mining. In addition, in the construction of an ecological environment evaluation system, most studies adopt the indictor-weight method, including AHP^19^, fuzzy evaluation method^20^, principal component analysis (PCA)^21^, support vector machine (SVM)^22^ and random forest method^23^. AHP can select different indexes and evaluation levels according to the characteristics of the regional ecological environment, but it has subjective factors. The fuzzy evaluation method is simple in the calculation but not sensitive enough in response to the ecological environment. PCA can objectively determine the weight of the evaluation index and avoid subjective arbitrariness. However, this method also has some information deficiencies. SVM can deal with nonlinear data well, but it is uncertain in the process of eco-environment evaluation and requires a large amount of memory and time for computation. The random forest method does not require the assumption of ostensive factors and could analyze the interaction between indicators, but it may overfit in some noisy classification or regression problems.

The projection pursuit model projects the high-dimensional data onto the low-dimensional space, constructs the objective projection function, and finds the best projection that can reflect the structure or features of the original high-dimensional data^24^. This method avoids subjective factors and masses of missing pieces, by which objective results can be obtained, and has achieved great application effect in water resource evaluation^25^, land use evaluation^26^ and watershed flood analysis^27^. Driving force analysis of evaluation results is also an important part of the eco-environment assessment. At present, most studies mainly analyze driving factors through linear regression fitting^28^, grey correlation analysis^29^ and other quantitative methods. However, it is pretty limited for statistical methods of spatial heterogeneity. The geographic detector is a statistical method that reveals all driving forces behind dependent variables by detecting spatial diversity, and could determine the quantitative interpretation capacity of independent variables to dependent variables by statistical analysis of spatial distribution similarity between independent variables and dependent variables, also could detect the interaction of two factors on the dependent variable.

Ibei Coalfield is one of the four largest coalfields in Xinjiang Province, China, and this is the unique oasis mining area. It has been observed that underground coal deposited in Ibei Coalfield are of shallow depth, great thickness and simple geological conditions. In the future, large-scale mining activity may lead to a sequence of environmental problems, potentially threatening the stability of the desert-oasis ecosystem. Therefore, it is necessary to make a quantitative scientific evaluation of the EEQ in this region, explore the driving factors of the eco-environment, and provide a reference for the subsequent eco-environment management and coal resources development.

Based on this, the research content of this paper is as follows: (1) Taking Ibei coalfield as an example, analyzing the natural geographical conditions, geological mining conditions and ecological environment conditions, constructing the indicators evaluation system of EEQ in the mining area. (2) Based on the advantages and objectivity of the projection pursuit model in solving high dimensional nonlinear data, combined with modern optimization algorithm (genetic algorithm), set up eco-environmental quality evaluation (EEQE) model, evaluated Ibei coalfield EEQ. (3) The study zoned the Ibei coalfield by spatial autocorrelation, analyzed driving factors of eco-environment by geographic detector model, discussed the response mechanism of each evaluation indicators to the eco-environment system of the mining area under the disturbance of the mining area. (4) Based on the above, this paper proposes the corresponding mining environmental governance plan.

## Study area

**Figure 1:**
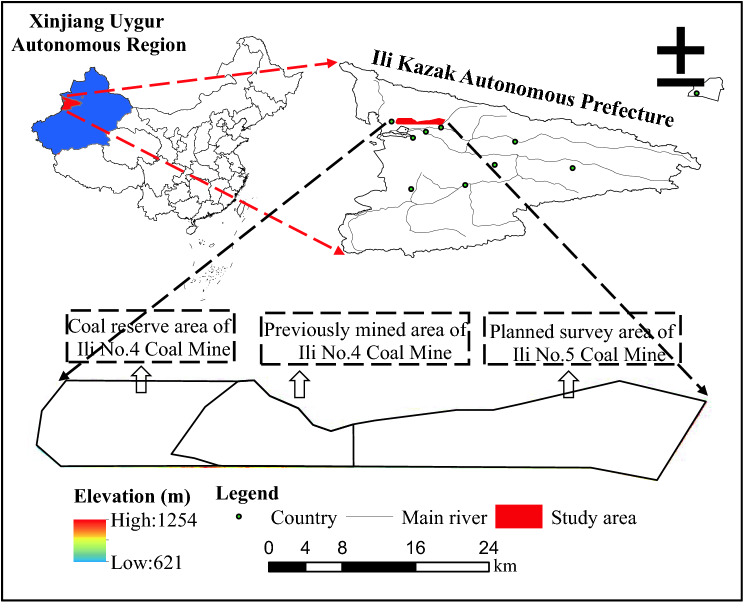
Location and elevation of the study area. The Figure is created using ArcGIS ver.10.2 (https://www.esri.com/).

The study area is in Ili Kazak Autonomous Prefecture, Xinjiang Province, China. As shown in Fig. 1, the area spans 31.1–50.0 km longitudinally and 5.6–11.1 km latitudinally, with a total area of about 450 km^2^. The study area is divided into three areas: the previously mined area of Ili No.4 Coal Mine, the coal reserve area of Ili No.4 Coal Mine, and the planned survey area of Ili No.5 Coal Mine. In the previously mined area of Ili No.4 Coal Mine adopts top-coal caving method. Due to the expanding mining scale year by year, the previously mined area of Ili No.4 Coal Mine is facing environmental problems such as vegetation destruction, soil erosion, and other environmental problems. The coal reserve area of Ili No.4 Coal Mine and the planned survey area of Ili No.5 Coal Mine are still in the mine planning stage, and there are only scattered industrial sites on the surface. The study area is located in inner Eurasia, determining its temperate semi-arid continental climate where the annual average temperature is 10.4 ℃, precipitation is 368.5 mm, and evaporation is 1500 mm. Tianshan is just on the east, giving rise to extremely uneven topography and deeply incised valleys. The maximum altitude difference of the area reaches 633 m. Regional surface water is mainly valley streams formed from meltwater and seasonal rainfall.

## Data and methodology

### Data collection and processing

To evaluate the EEQ of the study area, the following information needs to be extracted from the obtained data: terrain, climate, land, river system, population, vegetation, and mining area. The data used in this article are as follows: Landsat 8 Operational Land Imager (OLI) remote sensing image was acquired on August 7, 2020, which were used to extract normalized difference vegetation index (NDVI) and Land-use type. A digital elevation model (DEM) with a resolution of 30 m in the Geospatial Data Cloud was used to extract slope, aspect, and river system distance. Interpolation data of precipitation and evaporation in the study area in 2020 are from Yining Meteorological Bureau. The population data of the study area came from the statistical Yearbook of Xinjiang Uygur Autonomous Region in 2020. Geomorphology data were obtained from the Chinese Geomorphology Database, which was provided by the Institute of Tibetan Plateau, Chinese Academy of Sciences. Table 1 lists the collected data. Before further spatial analysis, these original data are uniformly processed and projected onto the WGS1984 coordinate system with ArcGIS software.

**Table 1:** Data collection and processing.

Data use	Data processing approaches	Source	Date type
Terrain	Extraction or calculation using DEM data	Geospatial Data Cloud http://www.gscloud.cn	ASTERDEM, spatial resolution 30 m
Landform	Cutting from vector map	National Tibetan Plateau Environment Data Centre http://data.tpdc.ac.cn/zh-hans/	
Climate	Interpolation of long-time average value using Kriging	Yining Meteorological Bureau	Monthly data of precipitation and evaporation
River system distance	Analyzing river system by DEM data and solving with Euclidean distance	Geospatial Data Cloud http://www.gscloud.cn	ASTERDEM, spatial resolution 30 m
Vegetation	Inversion of remote sensing satellite imagery	Geospatial Data Cloud http://www.gscloud.cn	Landsat 8 OLI image, spatial resolution 30 m, 2020/8
Land utilization	Interpretation of remote sensing satellite imagery	Geospatial Data Cloud http://www.gscloud.cn	Landsat 8 OLI image, spatial resolution 30 m, 2020/8
Population	Interpolation using Kriging	Statistical Yearbook of Xinjiang Uygur Autonomous Region	Population spatial distribution data
Coal mines	Data spatialization using ArcGIS	Field measurement	Mining, surface subsidence spatial data

### Evaluation methodology

**Figure 2:**
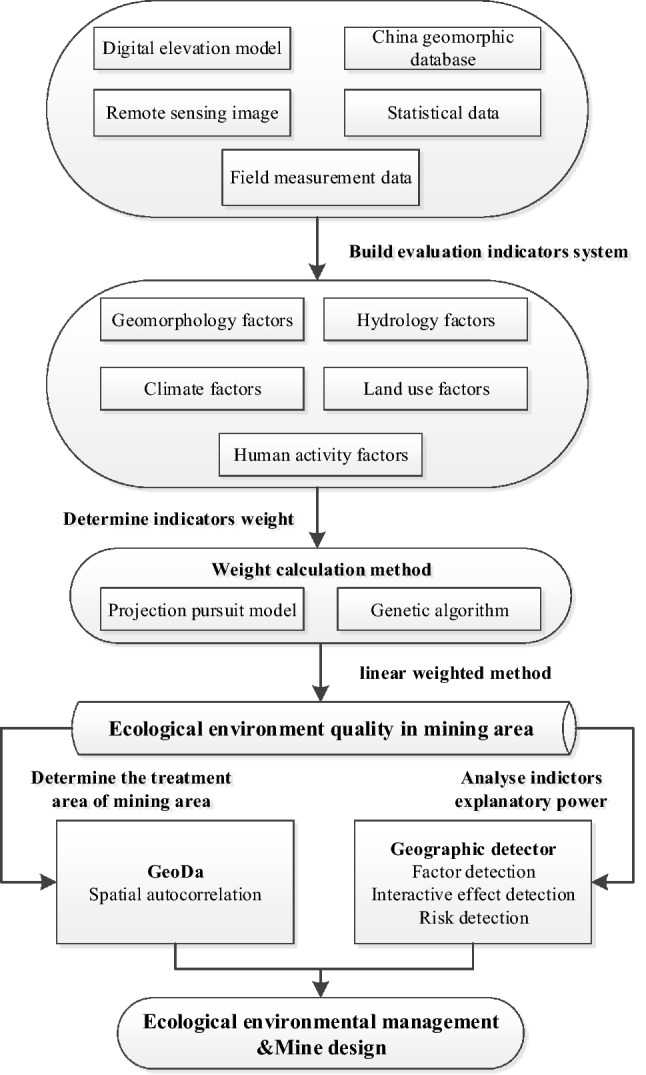
Research framework.

Figure 2 exhibits the procedures and methods of constructing an EEQE evaluation system of the mining area. We select the indicators that can reflect the ecological environment level of the mining area and then use the genetic projection pursuit model to calculate the weight of the indicators. After evaluating and mapping the regional ecological environment, the characteristics of EEQ and driving factors are analyzed by spatial autocorrelation and geographic detector to guide local environmental protection and mine design.

#### Indicator selection

Indicators for assessing the eco-environment levels of a coal mine are rather complicated, and an appropriate choice indicator is critical for EEQE. In accordance with the principle of evaluation indictor system construction, also taking practicality and accessibility into account, 13 indicators are selected in regard to geomorphology, climate, hydrology, land resource, vegetation, and human activity factors.

Geomorphology is closely related to hydrology, soil, vegetation, and creature; its impact on the ecological environment is characterized using elevation, terrain slope, terrain aspect and geomorphic type^30^. Annual average precipitation and average annual evaporation are used to describe regional climatic conditions. Comparatively, adequate rainfall results in higher vegetation coverage. In contrast, with greater the evaporation, the lower the moisture content of surface soils, possibly leading to water loss and land salinization^31^. Shallow aquifer is a key hydrological factor underpinning ecosystem stability and characterized by specific yield; the greater the specific yield, the stronger the ability of an aquifer in water release^32^. River system distance represents the situation of surface waters, reflecting the impact of flows on surrounding soil erosion^33^ and on the circumstance of flora and fauna community^34^. As a factor affecting water and soil conservation as well as the stability of the ecosystem, vegetation coverage is represented via NDVI^35^. Also, land resource utilization and layout are considered because of their eco-environmental impact; for example, the regional ecology of the land for construction can be damaged to a great extent^36^.

**Figure 3:**
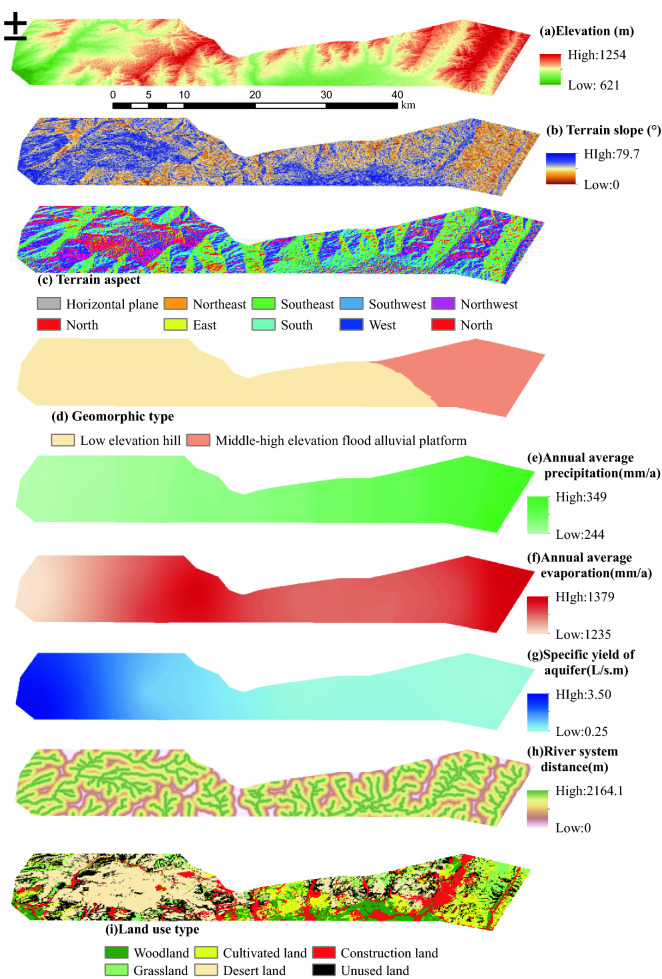

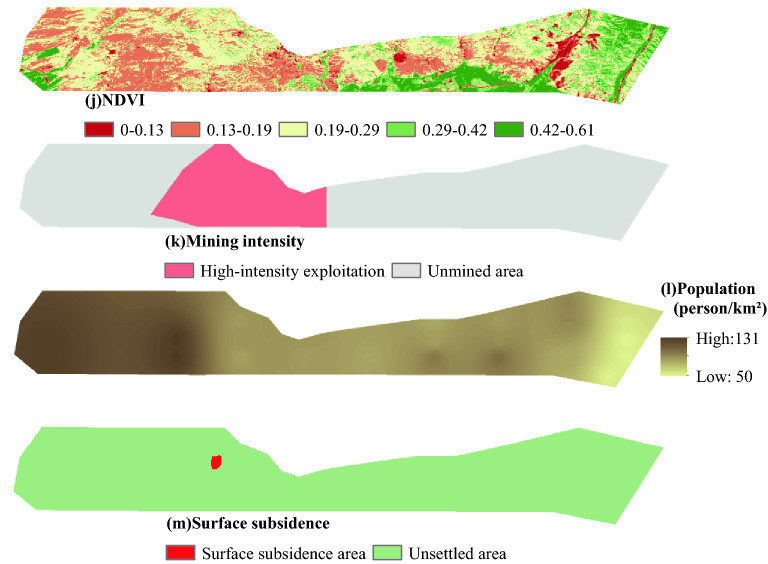
Eco-environmental quality evaluation indicators. The Figure is created using ArcGIS ver.10.2 (https://www.esri.com/).

In generally, the impact of underground coal mining on the ecological environment is positively correlated to mining intensity^37^. The indicators representing mining intensity include positive external indicators (listed in Table 2) and negative external indicators^38^. The negative ones refer to the consequences resulting from coal seam extraction, include overburden strata movement and eco-environmental damage. According to the classification standard, the previously mined area of Ili No.4 Coal Mine can be classified into the high-intensity mining area. In contrast, the coal reserve area of Ili No.4 Coal Mine and the planned survey area of Ili No.5 Coal Mine should be unmined area. Within the whole area of the case, surface subsidence induced by coal exploitation can decrease available land resource, accelerate soil erosion, alter runoff and catchment conditions, and deteriorate ecological environment. Figure 3 shows the data map of the 13 indicators.

**Table 2:** Positive external indicators of large-scale underground longwall mining.

Indicators	Parameter	Indicators	Parameter
Coal seam thickness	≥ 3.5 m	Mine output	500–1000 Mt/a, or ≥ 1000 Mt/a
Panel width	≥ 200 m	Ratio of depth to the thickness	*H*/*M* < 100
Retreat rate	≥ 5 m/d

#### Methods configuration

1. Genetic projection pursuit model
  The weight of 13 indicators is calculated using the genetic projection pursuit model. For this purpose, high dimensional data are projected onto a lower-dimensional space to construct objective functions and identify the best projection path capable of reflecting the structural feature of high dimensional data^39,40^. The configuration of the genetic projection pursuit model requires three procedures that are, data standardization, projection indicator function construction, and projection indicator function optimization.
  The first procedure is data standardization. The indicators are different in dimension and order of magnitude, which makes them lack comparability. The 13 indicators in this paper are divided into quantitative indicators and qualitative indicators. Quantitative indicators include elevation, slope, aspect, annual average precipitation, annual average evaporation, specific yield of aquifer, river system distance, NDVI, and population. The raster calculator in ArcGIS is used to normalize the data.
  For the indicators which have a positive correlation with the ecological environment, the equation is expressed as follows^41^_:_
  1 $$ X_{i} = [x_{i} - min(x_{i})]/[max(x_{i}) - min(x_{i})] $$
  For the indicators which have a negative correlation, the equation is:
  2 $$ X_{i} = [max(x_{i}) - x_{i}]/[max(x_{i}) - min(x_{i})] $$
  where $X_{i}$ is the normalized value of the variable $i$,$x_{i}$ is the initial value of the evaluation indicator.
  The qualitative indicators are graded according to the previous studies^42,43^. To determine the degree of influence of each indicator, We divided the qualitative indicators into five levels referring to the "five-equal division"^44^ method. Accordingly, $V = \{ V_{1},V_{2},V_{3},V_{4},V_{5}\}$ corresponded to the five grades: $V = \{ {\text{worse, bad, medium, good, better}}\}$. Assign a value to each grade to turn qualitative evaluation into quantitative evaluation, that is $V_{1} = 1$, $V_{2} = 2$, $V_{3} = 3$, $V_{4} = 4$, $V_{5} = 5$. Finally, we use the range method to normalize qualitative indicators. The evaluation grade of each qualitative indicator is evaluated by experts, and the evaluation grade results are shown in Table 3.

**Table 3:** Determination of evaluation grade of qualitative indicators.

Assessment indicators	Assessment grades				
	1	2	3	4	5
Geomorphic type		Middle-high elevation flood alluvial platform			Low elevation hill
Land use type	Construction land	Desert land	Cultivated land	Grassland	Woodland
	Unused land				
Mining intensity	High-intensity exploitation				Unmined area
Surface subsidence	Surface subsidence				Unsettled area
  After data standardization, the functions for projection indicators should be constructed. The sample-set is $\left\{ {x(i,j)\left| {i = 1,2} \right.} \right. \ldots ,n;\left. {j = 1,2 \ldots, m} \right\}$, where *m* refers to the number of evaluation indicators and *n* is the number of samples. The one-dimensional projection ($V_{i}$) of *m*-dimensional data along the direction $c = \left\{ {c(1),c(2)} \right.,c(3), \ldots ,\left. {c(m)} \right\}$ is expressed as:
  3 $$ V_{i} = \sum\limits_{j = 1}^{m} {c_{j}} \cdot x(i,j), \quad i = 1,2 \ldots ,n $$
  To meet two requirements that (i) local projection points should aggregate to the greatest extent and (ii) overall the projection should disperse as much as possible, a projection indicator function is established:
  4 $$ Q(c) = S(c) \cdot D(c) $$
  5 $$ S(c) = \sqrt {\frac{{\sum\nolimits_{i = 1}^{n} {(V_{i} - E(V_{i}))^{2} } }}{n - 1}} $$
  6 $$ D(c) = \sum\limits_{i = 1}^{n} {\sum\limits_{j = 1}^{n} {[R - r_{ij}]} } \cdot f[R - r_{ij}] $$
  where $S(c)$ is inter-class distance, $D(c)$ is within-class density, $E(V_{i})$ is the mean of $\left\{ {V_{i}} \right.\left| {i = } \right.1,\left. {2 \ldots ,n} \right\}$, $r_{ij}$ is inter-sample distance, $r_{ij} = \left| {V_{i}} \right. - \left. {V_{j}} \right|$, *R* is the window radius of local density, and $f[R - r_{ij}]$ is step function where it $R$ is greater than $r_{ij}$, $f[R - r_{ij}]$ equals one but if not, $f[R - r_{ij}]$ equals zero. $D(c)$ represents the aggregation level of projection points; much greater the value of $D(c)$, more aggregated the points.
  The primarily constructed functions can be further optimized. The change of projection index function $Q(c)$ is determined by projection direction $c$. Different projection directions can reflect different structural characteristics of data, so it is necessary to figure out the optimal projection direction. The maximized objective function and the corresponding constraint condition are expressed as:
  7 $$ Max{:}\;Q(c) = S(c) \cdot D(c) $$
  8 $$ s.t.\sum\limits_{j = 1}^{m} {c_{j}{^{2}} } = 1 $$
  Considering that the best projection direction calculation is a complicated nonlinear optimization problem, we use the genetic algorithm to identify the optimal projection direction. The steps are shown in Table 4.

**Table 4:** Steps for determining the best projection direction.

Steps	Operations
I	Generate X group of initial unit projection direction vectors randomly, and calculate the projection eigenvalues of each group according to Eq. (1)
II	According to Eqs. (5) and (6), we calculate the inter-class distance and within-class density in the projection direction of $X$ group respectively, and substitute it into Eq. (4) to calculate the objective function $Q(c)$ in the projection direction of $X$ group
III	According to genetic algorithm (selection, mutation and crossover) operation, generate several groups of new projection direction vectors
IV	According to Formula Eq. (6), since $R$ is a continuous data with spatial distribution, a new projection direction vector is selected from multiple sets of projection direction vectors only to meet the principle that the larger the maximum value of $R$ is, the better
V	Repeat the above operations until the maximum value of $Q(c)$ does not increase and the corresponding projection direction vector is the optimal projection direction. Each component of the optimal projection direction represents the contribution of the related indicator to the EEQ for the study area
2. Eco-environmental quality evaluation modeling
  Based on the optimal projection direction vector obtained above, the eco-environmental quality of the coal mining area is quantified using the mining area eco-environmental quality index (MAEEQI), in which the direction vector is as the weight of each evaluation indicator. The weighted summation of all indicators is calculated:
  9 $$ {\text{MAEEQI}} = \sum\limits_{i = 1}^{n} {w_{i} \cdot u_{i}} = w_{1}u_{1} + w_{2}u_{2} + \cdots + w_{2}u_{2} $$
  10 $$ u_{i} = c_{j}{^{2}} $$
  where $u_{i}$ represents the weight of each indicator, $w_{i}$ is the standardized value of each indicator, and $n$ is the number of evaluation indicators.
3. Spatial autocorrelation
  As an approach to analyze the distribution characteristics of data, spatial autocorrelation is helpful for testing the significance of an attribute value of variables and verifying the relevance of attributes between adjacent points. In this paper, spatial autocorrelation analysis is used to study the aggregation characteristics of the eco-environment conditions in Ibei Coalfield^45,46^.
  Global autocorrelation characterized the aggregation and dispersion degree of eco-environmental quality within the whole space and expressed using Global Moran's *I* ranging between − 1 and 1. There is:
  11 $$ I = \frac{{n\sum\nolimits_{i = 1}^{n} {\sum\nolimits_{j = 1}^{n} {w_{ij}(x_{i} - \overline{x} )(x_{j} - \overline{x} )} } }}{{\sum\nolimits_{i = 1}^{n} {\sum\nolimits_{j = 1}^{n} {w_{ij}(x_{i} - \overline{x} )^{2} } } }} $$
  where *I* is the indicator of global autocorrelation, n is the total amount of elements, *x*_*i*_ and *x*_*j*_ are the eco-environmental quality level of spatial unit *i* and *j* respectively, $\overline{x}$ is the average value of eco-environmental quality, and *w*_*ij*_ represents spatial weight coefficient matrix^47^.
  Further, local spatial autocorrelation (expressed as Local Moran's *I,* ranging from − 1 to 1) can be used to analyze the aggregation and dispersion of eco-environmental quality in a localized area. There is:
  12 $$ I_{p} = \frac{{n(x_{i} - \overline{x} )\sum\nolimits_{j = 1}^{n} {w_{ij}(x_{j} - \overline{x} )} }}{{\sum\nolimits_{i = 1}^{n} {(x_{i} - \overline{x} )^{2} } }} $$
4. Geographic detector
  As a statistical method to analyze the spatial heterogeneity of data, the geographic detector can identify the causality of different elements within a localized scale. The advantage is that this method can not only detect both quantitative and qualitative data but also determine the interactive effect of two factors on the dependent variable, even without any prior assumptions and constraint conditions^48,49^. Geographic detectors are divided into three detection methods, including factor detection, interactive effect detection and risk detection.
  Factor detection is used to identify the spatial heterogeneity of eco-environmental quality in Ibei Coalfield and to analyze the impact degree of various indicator factors (*X*) on eco-environmental quality (*Y*). The result is measured using *q* value, which can be expressed as:
  13 $$ q = 1 - \frac{{\sum\nolimits_{h = 1}^{L} {N_{h}\sigma _{h}{^{2}} } }}{{N\sigma^{2} }} = 1 - \frac{SSW}{{SST}} $$
  where *L* is the layer of variable *Y* or factor *X*, *N*_*h*_ is the number of units in the *h*th layer, *N* is the number of units in the whole area, $\sigma_ {h}{^{2}}$ is the variance of *Y* in the *h*th layer, $\sigma^{2}$ is the variance of *Y* in the whole area, $SSW$ and $SST$ represent the sum of the variance of one layer and the whole area, respectively. Figure 4 shows the principle of factor detection^50^.

**Figure 4:**
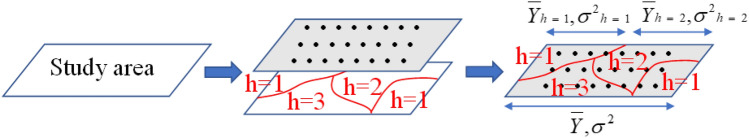
Fundamentals of factor detection.
  The principle of interaction detection is to identify the interaction between different indicators, that is, to evaluate whether the cooperation of evaluation indicators $X1$ and $X2$ will enhance or weaken the explanatory power of variable $Y$ (eco-environmental quality), or the influence of these indicators on $Y$ is independent.
  $q$ that is corresponding to a single indicator is calculated, and so does $q$ under the interaction of two indicators. Then comparisons are conducted among *q*, $q(X1 \cap X2)$, and the sum of $q$, and the results are divided into five categories. The types of interactions are shown in Table 5.

**Table 5:** Classification of interaction type.

Criteria	Interaction type
$q(X1 \cap X2) < \min (q(X1),q(X2))$	Nonlinear attenuation
$q(X1 \cap X2) > \max (q(X1),q(X2))$	Bilinear enhancement
$\min (q(X1),q(X2)) < q(X1 \cap X2) < \max (q(X1),q(X2))$	Single-linear attenuation
$q(X1 \cap X2) = q(X1) + q(X2)$	Mutual independence
$q(X1 \cap X2) > q(X1) + q(X2)$	Nonlinear enhancement
  Risk detection is to estimate whether the attribute means the value of two subareas has a significant difference, expressed as *t* statistics.
  14 $$ t(\overline{y} _{h = 1} - \overline{y}_ {h = 2}) = \frac{{\overline{Y} _{h = 1 }- \overline{Y} _ {h = 2}}}{{ \left[\frac{{Var(\overline{Y}_ {h = 1})}}{n_{h = 1}} + \frac{{Var(\overline{Y} _{h = 2})}}{n_{h = 2}} \right]^{1/2} }} $$
  where $\overline{Y} _{h}$ is the attribute mean value of area *h*, $n_{h}$ is the number of samples in the area, and $Var$ represents variance.

## Results

### Evaluation indicator weight

The calculation of indicator weight is achieved using Matlab programming, with the total process including the optimization solution of the model via genetic algorithm and then cross iteration. The initial population size of the parent generation is 400, the crossover probability is 0.8, the mutation probability is 0.3, and the number of excellent individuals is 200. The optimized direction vectors obtained via the above procedures are the weight of various indicators, as listed in Table 6.

**Table 6:** Indicators weight.

Indicators	Weights	Indicators	Weights
Elevation (X1)	0.026	River system distance (X8)	0.086
Terrain slop (X2)	0.027	Land use type (X9)	0.075
Terrain aspect (X3)	0.069	NDVI (X10)	0.084
Geomorphic type (X4)	0.076	Mining intensity (X11)	0.089
Annual average precipitation (X5)	0.125	Population density (X12)	0.070
Annual average evaporation (X6)	0.076	Surface subsidence (X13)	0.121
Specific yield of aquifer (X7)	0.076		

### Eco-environmental quality feature

Based on the above-calculated weights of 13 indicators, an EEQE model for Ibei Coalfield can be obtained:

15 $$ \begin{aligned} MEEQI & = \sum\limits_{i = 1}^{13} {w_{i} \cdot u_{i}} \hfill \\ & = 0.020w_{1} + 0.027w_{2} + 0.068w_{3} + 0.076w_{4} + 0.084w_{5} + 0.125w_{6} + 0.076w_{7} \hfill \\ & \quad + 0.076w_{8} + 0.072w_{9} + 0.070w_{10} + 0.089w_{11} + 0.121w_{12} + 0.086w_{13} \hfill \\ \end{aligned} $$

For the mining area, its eco-environmental quality index are determined by Eq. (15). Further conformity and classification are successively conducted on the indicators by using ArcGIS analytical tool, followed by visualization of mining area eco-environmental quality index (MAEEQI) by combining Eq. (15) and weighted summation tool. The standard adopted in this paper for eco-environmental quality evaluation and grading sources from Technical Criterion for Ecosystem Status Evaluation and currently existing research results^51,52^. The EEQ of the study area is graded into five levels from worse to better by using natural break (Jenks), with results listed in Table 7.

**Table 7:** Mining area eco-environmental quality evaluation and grading.

Grade	Worse	Bad	Medium	Good	Better
Numerical scope	< 0.501	0.501–0.552	0.552–0.606	0.606–0.668	> 0.668

**Figure 5:**
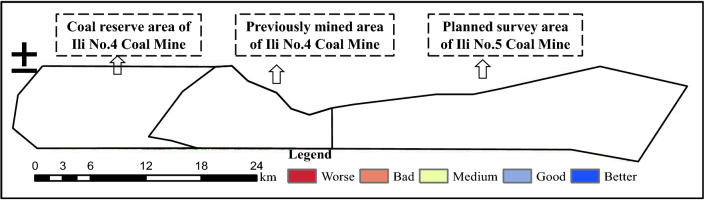
Eco-environmental quality evaluation and grading. The Figure is created using ArcGIS ver.10.2 (https://www.esri.com/).

In Fig. 5, the area with bad and worse eco-environmental quality accounts for 40.4% of the whole area, the regions of the other three levels are 59.6% (Table 8). Overall, the eco-environmental quality of Ibei Coalfield tends to be the medium level. Compared with the districts of Ili No. 4 Mine, the EEQ of the planned survey area of Ili No.5 Coal Mine is better, especially in the south and east of the planned survey area as there is sufficient precipitation and greater vegetation coverage. In addition, No. 5 Coal Mine is still under planning, currently without high-intensity coal mining activity and strong engineering disturbance on the local ecology and environment. However, the middle area of Ili No. 5 Coal Mine exhibits bad to worse eco-environmental conditions for urbanization and human activity.

**Table 8:** Statistical area and proportion of each grade.

Grades	Area (km^2^)	Proportion (%)
Worse	66.56	12.3
Bad	151.98	28.1
Medium	165.66	30.7
Good	98.82	18.3
Better	54.58	10.6

**Figure 6:**
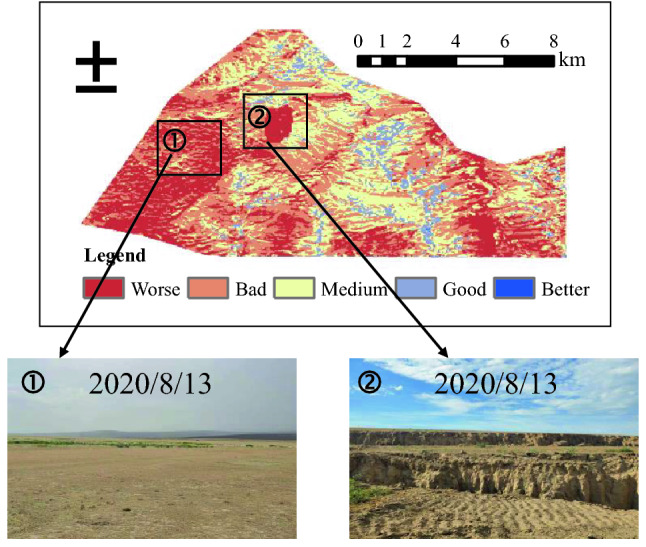
Eco-environmental quality grading for Ili No. 4 Coal Mine and eco-environmental problems, ➀ Surface subsidence and ➁ Vegetation degradation. The Figure is created using ArcGIS ver.10.2 (https://www.esri.com/).

The overall eco-environmental quality of No. 4 Coal Mine is not as good as No.5 Coal Mine (Fig. 6); the worse area is up to 68.5% of the total No. 4 Coal Mine area, and good to better areas only account for 5.6% (Table 9). Up to now, the No. 4 Coal Mine has worked shallow longwall mining for many years, causes a series of eco-environmental problems such as localized surface subsidence (Fig. 6) and vegetation degradation. Despite taking land and environmental treatment, mining impact is more than the intrinsic eco-environmental bearing capacity, mining-induced damage is faster than ecological rehabilitation.

**Table 9:** Statistical area and proportion of each grade of the previously mined area of Ili No.4 Coal Mine.

Grades	Area (km^2^)	Proportion (%)
Worse	33.48	28.4
Bad	46.45	39.6
Medium	31.12	26.4
Good	5.83	4.96
Better	0.75	0.64

In the coal reserve area of Ili No.4 Coal Mine, about 51.1% of the area is graded into medium to better, indicates a critical state. In this area, low-altitude hilly topography prevails, with large population density, scattered grasslands and low vegetation coverage. Environmental treatment and appropriate mine arrangement have just begun. Some infrastructural project for underground mining activity in the future leads to eco-environment deterioration, which should be a focus for the follow-up engineering operations.

### Spatial pattern of eco-environmental quality

**Figure 7:**
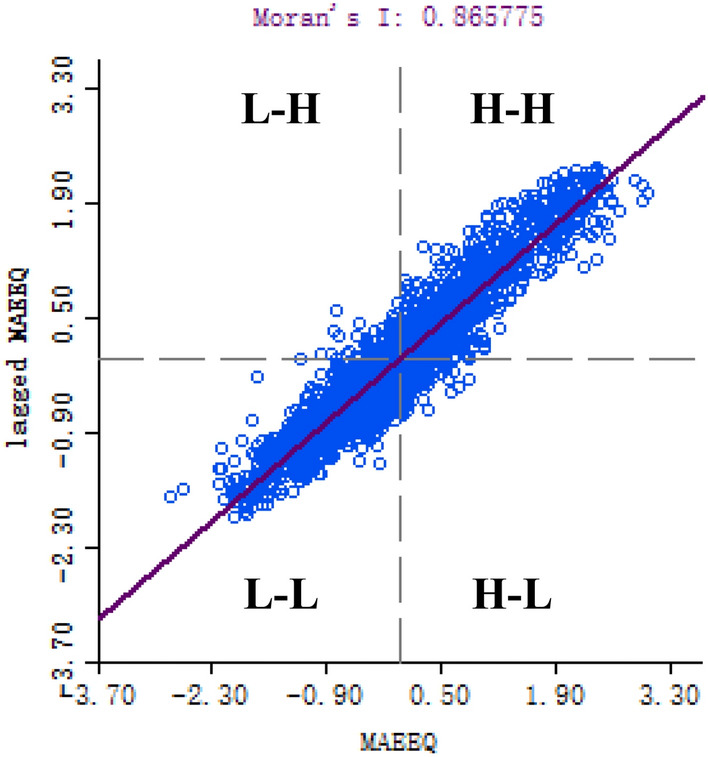
Moran scatter diagram of mining area eco-environmental quality (MAEEQ).

**Figure 8:**
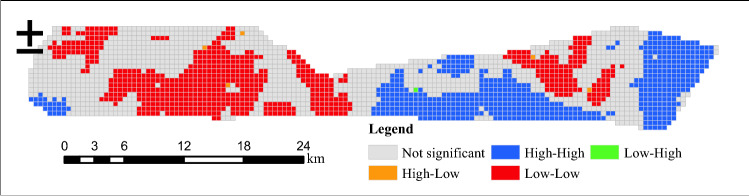
LISA aggregation diagram of eco-environmental quality. The Figure is created by GeoDa ver.1.20 (http://geodacenter.github.io/).

Under the premise that the scale information is complete and the evaluation result is accurate, the eco-environment feature of the study area is graphically resampled using 500 × 500 m grid meshes, totally outputting 2294 sampling points. Then the spatial autocorrelation of eco-environmental quality is computed using GeoDa. GeoDa provides a user-friendly interface and a wealth of methods for exploratory spatial data analysis, such as spatial autocorrelation statistics and basic spatial regression analysis. The results pass the 95% confidence test and are plotted into Moran scatter diagram (Fig. 7) and LISA index spatial aggregation diagram (Fig. 8). The calculation shows that the Moran's *I* of Ibei Coalfield equals 0.865, suggesting a significantly positive correlation in eco-environmental quality and distinguished spatial aggregation phenomenon and that the area with low eco-environmental quality can impact the adjacent area.

In Fig. 7, most data points are scattered along the regression line, featuring an eco-environmental quality pattern that homogeneity aggregates and heterogeneity disperses. Combining the spatial adjacency characteristics, shows that two quadrants H-H and L-L exhibit spatial heterogeneity; in H-H and L-L the data points are concentrated, indicating that the area with high eco-environmental quality and area with low-quality aggregate separately and show a significantly positive correlation. Differently, in quadrants H-L and L-H the data points are rather lesser and scattered and witness negative correlation, indicating that high eco-environmental quality area and low-quality area are surrounded mutually.

It is analyzed from Fig. 8 that H-H aggregation takes 574 spatial units in the southwest of the coal reserve area of Ili No.4 Coal Mine, southwest and east of Ili No.5 Coal Mine. L-L aggregation takes 661 spatial units mainly in the previously mined area of Ili No.4 Coal Mine and partial area of the coal reserve area of Ili No.4 Coal Mine, and further coal mining activity can deteriorate the ecology and environment of these two areas. Meanwhile, some regions have outliers of eco-environmental quality, which is represented by H-L outliers. The outliers are mainly the grassland and forest surrounded by construction land and desert, which makes them exhibit higher quality than their surrounding areas. L-H aggregation only takes one spatial unit, thus showing weak autocorrelation and random distribution.

### Eco-environmental driving factors

The geographic detector model requires independent variables to be type data and dependent variables to be numerical data, so the zoning grades and zoning methods may affect detection results. To decrease errors, various indicators are zoned using the natural break (Jenks) method, giving rise to nine types in terms of surface elevation, terrain slope, terrain aspect, NDVI, annual average precipitation, annual average evaporation, aquifer specific yield, population density and river system distance, six types for land utilization, and two types for mining intensity, surface subsidence and landforms.

#### Driving factor detection analysis

**Figure 9:**
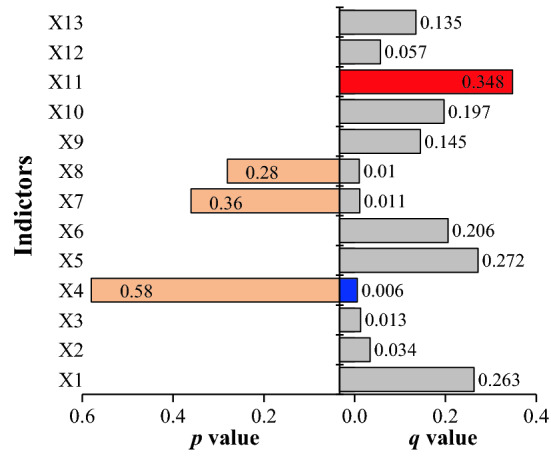
Detection results of various indicator factors.

Figure 9 shows the eco-environmental factors detected in Ibei Coalfield. The *q* value can reflect the difference of various indicators in driving local ecology and environment. The *p*-value corresponding to the *q* value of each type variables X (independent variable) in the geographic detector represents the statistical significance of this factor. Mining intensity (X11) ranks the first in terms of the interpretation ability, followed by annual average precipitation (X5), surface elevation (X1), annual average evaporation (X6), NDVI (X10), land use type (X9), surface subsidence (X13), population density (X12), terrain slope (X2), terrain aspect (X3), aquifer specific yield (X7), river system distance (X8) and landform type (X9). The current eco-environmental quality in Ibei Coalfield results from the interaction of natural conditions and human activities. If checking the interpretation ability, mining intensity is the primary factor driving the change of eco-environment level, with interpretation ability greater than 30%. Annual average precipitation, surface elevation and annual average evaporation are higher than 20%, and NDVI, surface subsidence and land use type are higher than 10%. The other indicators are of relatively weaker ability in interpretation. Aquifer-specific yield, river system distance, and landform type have high *P* values, so the impact is weak.

#### Indicator interaction analysis

**Figure 10:**
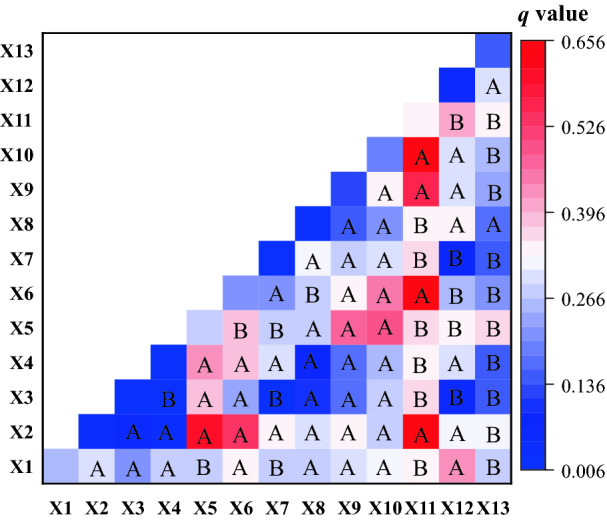
Detection results of indicator interaction: (**A**) refers to nonlinear enhancement; (**B**) refers to the linear enhancement.

The results of interaction detection are shown in Fig. 10. Figure 10 shows the interaction of terrain, climate, hydrology, land utilization, vegetation coverage and human activity is stronger than any single factor among them, suggesting that various factors are closely related and interactively affect coal mine eco-environmental quality. The interactive effect of various indicators on eco-environmental quality includes two forms, linear enhancement and nonlinear enhancement, and the forms account for 35.9% and 64.1% respectively. In detail, the combination of mining intensity and other indicators has stronger interpretation ability; X11 ∩ X10 (mining intensity and NDVI) shows the highest interpretation ability (*q* = 0.655), indicating that mining activity enhances the interpretation ability of NDVI as the single independent variable. The interaction of mining intensity (X11) with indicators including NDVI (X10), terrain slope (X2), annual average evaporation (X6) and land utilization (X9) sees a nonlinear enhancement effect, whereas sees linear enhancement with other indicators. In addition to mining intensity, the interaction of terrain slope (X2), annual average precipitation (X5) and annual average evaporation (X6) with other indicators also enhance their ability in interpreting the eco-environmental quality of the study area. Overall, the interactive effect of various factors on eco-environmental quality cannot be considered a simple superposition but a linear or nonlinear enhancement.

#### Impact of linearity and nonlinearity of indicators

**Figure 11:**
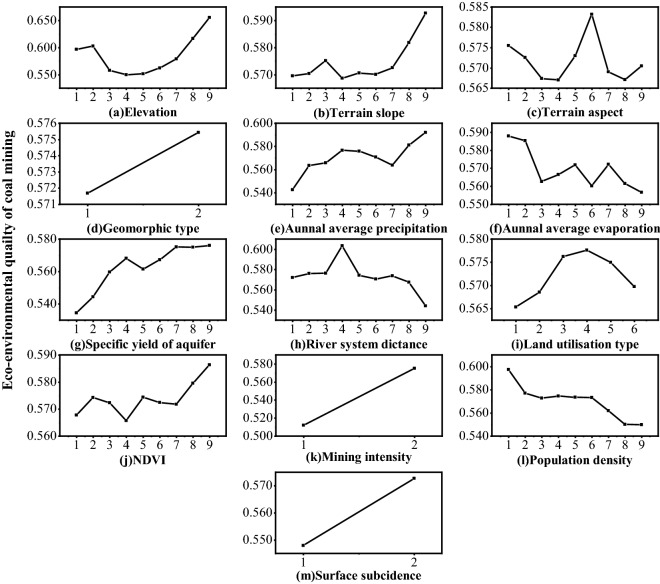
Change of mining area eco-environmental quality with indicator grades.

Risk detection can reflect the linear and nonlinear variation of mining area ecology and the environment with a specific indicator. As shown in Fig. 11, the horizontal axis represents the zone levels of each indicator. The eco-environmental quality changes linearly with the zoning grades of geomorphology, mining intensity and surface subsidence, but nonlinearly with other indicators.

Surface elevation and terrain slope share similar nonlinear characteristics; with the increase of zoning levels, mine eco-environmental quality experiences a decrease-increase process, as shown in Fig. 11a,b. In terms of terrain aspect, the less sunny side of a slope tends to show higher eco-environmental quality because of lesser evaporation and stronger ability to retain moisture, as shown in Fig. 11c. Figure 11e,g,j show wavelike increases in eco-environmental quality with annual average precipitation, aquifer specific yield and NDVI, indicating sufficient precipitation, shallow water resource and high vegetation coverage are conducive to the development of ecology and environment. Annual average evaporation and population density show a negative effect, indicated by Fig. 11f,l. Also, Fig. 11h,i exhibit that eco-environmental quality is closely related to river system distance and land utilization; when the area is 458.9 to 618.4 m away from a river and used as forestry land, it shows higher eco-environmental quality.

## Discussion

### Spatial pattern and driving factors of eco-environmental quality

Previous studies mainly focused on the impact of natural and human factors on the ecological environment of mining areas and seldom considered the driving factors and the interaction among factors on the same spatial scale. Therefore, this paper combined the factors of topography, meteorology, hydrology and land use, integrated the mining parameters into the mining intensity, and added them into the evaluation system. Through GIS, spatial autocorrelation and geographic detector, we not only studied the characteristics of EEQ in Ibei coalfield but also analyzed the driving factors hidden in detail.

**Figure 12:**
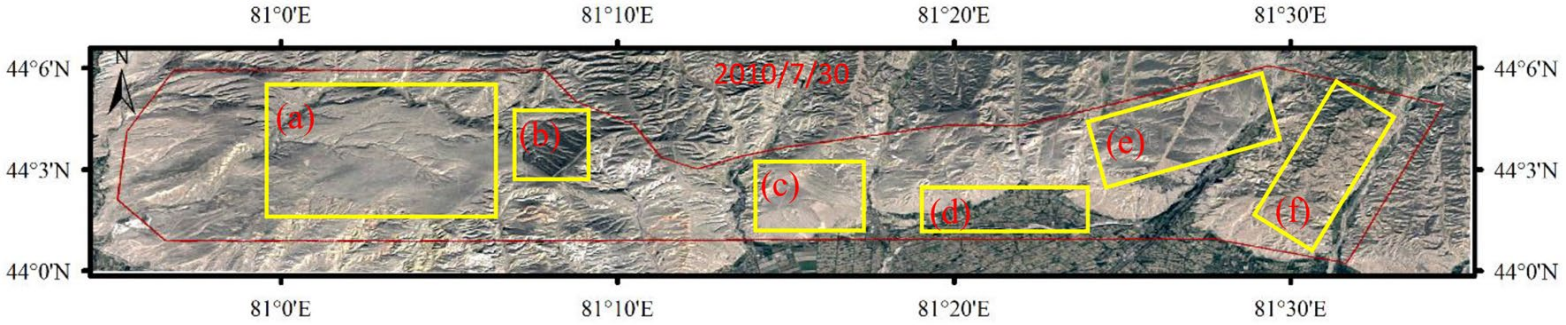
Google satellite image of Ibei coalfield before mining. The Figure is created using Google Earth Pro ver.7.3.4 (http://www.googlediqiu.com/).

In terms of the spatial pattern, the previously mined area of Ili No.4 Coal Mine has the worst ecological environment quality, followed by the coal reserve area of Ili No.4 Coal Mine and the planned survey area of Ili No.5 Coal Mine. In the whole study area, elevation and annual average precipitation show a gradient descent from east to west. Average annual precipitation is positively correlated with elevation, and more precipitation occurred in high-altitude areas and less in low-altitude areas. Sandy saline-alkali land is widely distributed in the study area, which seriously affects the growth of vegetation. In general, the distribution of NDVI is consistent with the average annual precipitation and elevation, while annual average evaporation and aquifer specific yield increase from east to west. The inhomogeneous distribution of these geomorphological, climatic and hydrological indicators should be the primary cause for the spatial heterogeneity of eco-environmental quality. Google satellite images before planning and mining were selected, as shown in Fig. 12. Google image clearly reflects the spatial heterogeneity of EEQ in the study area. We selected five representative regions in Fig. 12, area (b) and area (d) have high vegetation coverage and a better ecological environment. Areas (e) and (f) are dominated by grassland, with large slope changes and little human disturbance. Its ecological environment is second only to the area (b) and area (d). The soil type of region (a) and region (c) is sandy land, with little vegetation coverage and a poor ecological environment.

**Figure 13:**
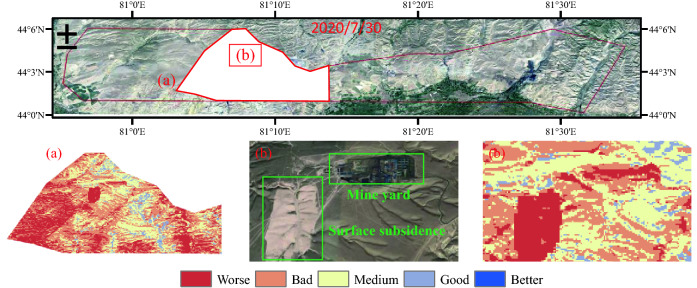
High mining intensity area (**a**), surface subsidence area (**b**) and corresponding eco-environmental quality characteristics. The Figure is created using Google Earth Pro ver.7.3.4 (http://www.googlediqiu.com/).

Mining intensity is the primary factor for the change of eco-environment level in coal mining area, with q value reaching 34.8%, in accord with the results obtained by Yang et al.^53^. The mining intensity of the previously mined area of Ili No. 4 Coal Mine is much higher than the coal reserve area of Ili No.4 Coal Mine and the planned survey area of Ili No.5 Coal Mine. High-intensity mining will affect the growth of surface vegetation and cause serious damage to the ecological environment, thereby affecting the spatial distribution of ecological quality, as shown in Fig. 13a. In addition, the surface subsidence alters the integrity of the earth's surface, accelerates soil erosion and negatively affects the ecological environment. However, the surface subsidence in the previously mined area of Ili No. 4 Coal Mine has been controlled, just a small part of the surface subsidence exists, where it has small spatial distribution, as shown in Fig. 13b. So the response of the ecological environment to surface subsidence is not obvious. Land-use type ranks second only to NDVI in explaining the ecological environment. The study area is mainly sandy land, and the construction land is mainly a mining industrial site, which significantly affects the soil and water conservation function of the land.

The natural factors have a significant impact on eco-environment levels. The ability of surface elevation, annual average precipitation and annual average evaporation to interpret eco-environmental quality is 26.3%, 27.2% and 20.6% respectively. In general, high elevation region tends to be cold, topographically fluctuated and with low soil quality, which has a great impact on the local ecological environment. However, in this study, eco-environmental quality enhances with elevation. It is speculated that high elevation regions experience less disturbance from human activities such as underground coal mining and construction projects. In addition, there can be a threshold for the elevation impact on ecological environment^49^; considering that the case area is not in the extremely high altitude area, the case area does not reach the threshold. Ibei Coalfield is in the northwest inland of China and less affected by monsoon, featuring greater evaporation than precipitation. Because ecological environments are relatively sensitive to rainfall^54^, annual average precipitation has a stronger interpretation ability than annual average evaporation. NDVI is closely related to the ecological environment. Areas with high vegetation coverage can resist rainfall erosion and other physical erosion, which has a positive promoting effect on the ecological environment, so its explanatory ability is also strong.

### Mining area eco-environment management strategy

The above conducted spatial autocorrelation analysis helps to identify the region that needs to be managed, and the geographic detector analysis indicates the factors driving the change of eco-environment. By combining the two analyses, an appropriate strategy for coal mining area eco-environment management is developed.

The low eco-environmental quality area (L-L) is classified as the management area, requiring multi-layer and intensive treatments such as ecological rehabilitation. The government should develop the corresponding protective strategies to motivate and compensate the coal mines by implementing eco-environmental protection policies^55^. Also, coal enterprises should control the intensity of land utilization and building density, and strengthen green infrastructure design, for example, filling topsoil cracks and growing special plants with drought tolerance since developed roots to prevent water and soil erosion. In addition, the core of scientific mining is to control the level of subsurface water since damaged plants and soil can restore in the short term by implementing land rehabilitation, declined water levels may need a longer period to recover^56^. From the engineering point of view, the groundwater level can be regulated by adjusting the mining parameters so as to protect the ecological environment. For example, reducing mining height is effective in preventing mining-induced fractures from reaching the shallow aquifer. Increasing the mining speed can reduce the permeability of overlying strata and maintain the groundwater level. Similarly, protective mining measures are also available, such as filling mining^57,58^ and slice mining^59^. Filling mining can effectively prevent surface subsidence. Slice mining can ensure the water isolation performance of aquicludes and slow down the water loss of aquifer. However, how to adjust the mining parameters and mining methods to maximize the output of coal resources while protecting the mining environment will be the key research in the future.

H-H area has better eco-environmental quality. Considering that the future coal mining activity may lead to localized deterioration, the H-H area is classified into a close attention area. On the one hand, coal enterprises should consider the eco-environment situation and combine the "3S" technique to construct an eco-environmental database and update by conducting continuous field measurement. On the other hand, new mines can take references from previous ones in production, implement the appropriate pre-mining design, and strengthen technical innovation over eco-environmental management.

Anomalous areas L-H and H-L can be used to predict and prevent eco-environmental risks as the localized low quality may propagate to the surrounding under the effect of spatial polarisation. So the areas labeled with L-H and H-L are classified into the protective area. In protective areas, it is necessary to designate a warning line for eco-environmental protection, required to preserve the grassland and forest, control urbanization rate and optimize land utilization patterns. Also, the above-designed policies are accompanied by financial support from the local government to construct an accountability strategy with the cooperation of government and coal enterprises.

### Strength and limits

By adopting the projection pursuit model, the paper analyses the data with high dimension, nonlinearity and nonnormal index to identify the optimal projection direction, which effectively solves the problem regarding complicated structures and features of high dimensional data in a nonlinear system. Compared with the conventional approaches like AHP, fuzzy comprehensive evaluation and PCA, this method analyses with data more objectively and avoids subjective factors^60^ and masses of missing pieces, thus to get the objective results. Further, the geographic detector method is of significant strength in analyzing the driving factors of eco-environmental quality variation; it can not only quantify the interaction within a single indicator and between various indicators but also reflect the nonlinear relationship between indicators and results if compared with conventional statistical analyses. In addition, the mining area eco-environment quality evaluation (MAEEQE) method developed in this paper can be relatively flexible in designing evaluation procedures and selecting evaluation indicators. The MAEEQE method can provide strategic guidance for ecological protection and environment management, allowing managers to conduct reasonable pre-mining design and post-mining rehabilitation.

The research also has some limitations. For evaluation indicator and model selection, it is unavoidable to be impacted by noisy data and data incompleteness; for example, it is pointed out that soil pattern can also affect the localized ecological environment to some extent^61,62^, which is not included into in this paper considering the difficulty in relevant data gathering. In addition, the evaluation on some systems, especially the terrestrial ecosystem, mainly takes normalized differential vegetation index (NDVI) and dominant plant community as the representative indicator, possibly lacking the indicators capable of reflecting the dynamic variation of a community, such as population competition. At present, identifying indicators that represent the dynamic process of eco-environmental quality fluctuation and its tendency is still a key study.

## Conclusion

Aiming to scientifically and accurately evaluate the eco-environmental quality of mining areas, we propose a new method that can quantitatively assess the eco-environmental quality. 13 indicators can reflect the ecological environment of mining area are selected from five factors, including topography, geomorphology, climate, hydrology, land use and human activities, and the ecological environment evaluation system of the mining area is improved. On this basis, combining genetic algorithm with projection pursuit model, an evaluation model of ecological environment quality in mining area is established, which can objectively evaluate the ecological environment quality in the mining area. The results show that the high-intensity mining area (previously mined area of Ili No.4 Coal Mine) has the worst ecological environment quality, followed by the coal reserve area of Ili No.4 Coal Mine and the planned survey area of Ili No.5 Coal Mine. Secondly, taking the eco-environmental quality evaluation results as dependent variables and 13 indicators as independent variables, the driving factors are analyzed by using the geographic detector. The results of geographic detector analysis indicate that both natural and human factors have a significant impact on the quality, one of the human factors about mining intensity shows the strongest ability to interpret the eco-environmental quality situation. The impact of two interactive indicators is greater than that of a single indicator, and there is a nonlinear relationship between each index and the ecological environment. Spatial autocorrelation analysis shows that Moran's I of the whole study area equals 0.865; eco-environment represents positive correlation and is of significant concentration and spatial heterogeneity feature. With the H-H, L-L, H-L and L-H in the LISA aggregation diagram as references, three areas were designated, including management area, close attention area and protective area, for which three different strategies are designed considering the results of driving factor analysis. This study provides a possible method for quantifying the ecological environment in the mining area and quantifying the driving forces of the ecological environment in the mining area. More importantly, it is necessary to advocate for comprehensive multifactor measures based on the intensity of the interaction between indicators.
